# Violent Exercise

**Published:** 1870-04

**Authors:** 


### VIOLENT EXERCISE.

COMPETITIVE exercises and games involving muscular effort are liable to cause life-long injuries and even sudden death. Within a few months, a promising youth died in a few days after a college rowing match. In an article on the contest in English waters between the representatives of an American and British university, occasion was taken to discourage such competitions, not only on the ground of their physical danger, but from their tendency to encourage drinking and gambling. Very recently the *London Times* says that "boat races are fast degenerating into betting traps, and such exhibitions should be removed from London waters." Base-ball, foot races, whether of walking or running, swimming matches, and all other forms of exercise which stimulate to the excessive employment of muscular effort, are alike unphilosophical, pernicious, and dangerous to life.

Athletes and prize-fighters do not often live long; the training process of itself is a strain upon the constitution and the powers of life. All violences and shocks to the human body are as certainly injurious as such things would be to any machine made by human hands. Many persons think that the " exposures " to rain and storm, and hardship, tend to

" HARDEN THE CONSTITUTION,"

led to such a belief from having observed that sometimes persons who have encountered these hardships have lived to old age in good health; such a result follows their large enjoyment of the healthful influences of out-door air and the necessity of living on plain food, and even that not always over abundant. Very much has been ignorantly written and credulously received as to the beneficial effects of the reaction of

" SHOWER-BATHS."

Except for a single occasion, as a means of rousing the system under the supervision of an intelligent physician, it may well be questioned whether any human being was ever benefited by a cold shower-bath or a dash of cold water on the person; it is, however, undeniably true that multitudes have been injured by them. Young children have been often thrown into epileptic convulsions by these operations, to die subsequently in an idiotic condition, as a very natural physiological result. Throwing cold water on infants, even by sprinkling, even for a single time, endangers convulsions; and as for making it a regular thing, it is a heartless cruelty; it is murderous.

The persons who live longest and who are most successful in business are those who live equably, whose physical movements, whose mental decisions, are the result of deliberation. Equability of mind, of temper, of bodily effort, should be cultivated by all. A walk of five miles in a day, will do an incomparably greater good than a race of the same distance within half an hour. The human constitution is no more hardened or improved by hardships or exposure to wind and storm, than a new hat or new coat is made better by being banged about. Dr. A. K. Gardner, in one of a series of admirable articles on health subjects in *Frank Leslie's Illustrated Weekly Newspaper*, says with force and truth under the heading

"HEART DISEASE,"

as a natural result of the more stimulated condition of the people of this whole country, he personally noticed two distinct cases of heart disease as the result of the "double quick," and prolonged and hurried retreat at the —

BATTLE OF BULL RUN.

In the same article, the fact of the many sudden deaths among the business men of New York, its bankers, its brokers, and its merchants, is explained by the hurried life of the times. He says, with undeniable truth, —

"The physical exertion of a broker's clerk in Broad Street one day is enough to develop heart disease. Watch one rush out from the heated Goldroom, without overcoat, run across the street, dodging men and vehicles, leaping, three steps at a time, up a couple or more flights of stairs; for five minutes in another hot room, writing with a frantic zeal; then again to dash out through rain, and sleet, and mud, to shout and halloo like a madman in the seething, steaming, tumultuous Board for a couple of hours, with interlarded repetitions of the recounted visits to his own or others' offices! How different is it from a Bull Run flight?

"Think, too, of the emotions of the present. See the heart jealousies of society, the family discords produced by ill-assorted marriages, family feuds, disputes about religious differences, free love, spiritual manifestations and affinities, the thirst for gold and fashion, and then do you wonder that the main organ of the body — which, by its action, furnishes the nutriment to produce all these violent physical actions, all these extravagant passions, that beats the time to which these actions go — do you wonder that there is so much heart disease?"

To all, to the young especially, it is earnestly advised as an essential element in making human life a grand success, with a large exemption from the discouragements and mishaps which make it a misery to multitudes, to cultivate, sedulously cultivate, the habit in physical efforts and in mental decisions,

DELIBERATION.

